# Nutrition regulates sex expression in a gender diphasy plant, *Lilium concolor* var. *megalanthum*

**DOI:** 10.3389/fpls.2023.1252242

**Published:** 2023-09-12

**Authors:** Xin Chen, Lei Wang, Xingfu Yan, Zhanhui Tang

**Affiliations:** ^1^ State Environmental Protection Key Laboratory of Wetland Ecology and Vegetation Restoration, Key Laboratory for Vegetation Ecology, Ministry of Education, School of Environment, Northeast Normal University, Changchun, China; ^2^ Key Laboratory of Ecological Protection of Agro-pastoral Ecotones in the Yellow River Basin, National Ethnic Affairs Commission of the People’s Republic of China, College of Biological Science and Engineering, North Minzu University, Yinchuan, China

**Keywords:** *Lilium concolor* var. *megalanthum*, growth and reproduction strategy, sexual system, nutrient availability, gender diphasy

## Abstract

**Introduction:**

The evolution and maintenance of plant polymorphism have always received much attention. Gender diphasy is a rare sexual system. Plant individuals with gender diphasy can adjust the resource allocation of different functional organs according to the changes of environmental conditions to regulate the sex expression of individuals, and the sex expression can be converted between years. However, our understanding of sex expression in plants is still very insufficient. In this study, we explored whether the perennial plant *Lilium concolor* var. *megalanthum* has a gender diphasy system and whether environmental resource availability affects its resource allocation and sex expression.

**Method:**

By collecting the bulbs of two sexual phenotypes (male and hermaphrodite) in the field and simulating the application of different levels of nutrients under the same habitat conditions, the growth and reproduction indexes and sex expression of plants in two years (2021 and 2022) were measured to evaluate the resource allocation strategy and sex expression pattern of *Lilium concolor* var. *megalanthum*.

**Results:**

We found that the sex expression of *Lilium concolor* var. *megalanthum* was variable in continuous years. Under limited resources, *Lilium concolor* var. *megalanthum* increases the biomass resources of the leaves and has a longer flowering period. Resource availability regulates the growth, reproduction and sex expression of *Lilium concolor* var. *megalanthum*. Bulb size is the main factor affecting its growth, reproduction and sex expression after accumulating sufficient resources.

**Discussion:**

This study confirms that *Lilium concolor* var. *megalanthum* has gender diphasy system. There is a strong trade-off between growth and reproduction under limited resources. Nutrient levels can regulate the reproduction and sex expression process of *Lilium concolor* var. *megalanthum*. With the growth of *Lilium concolor* var. *megalanthum* in consecutive years, the size dependence of bulbs may be the decisive factor in its sex expression.

## 1 Introduction

In the process of sexual reproduction of perennial flowering plants, the phenomenon that individual sex changes (transforms) between consecutive years is called gender diphasy system (Lloyd, 1984). Gender diphasy is a very rare sexual system in nature and only exists in a few plant species (Schlessman, 1991). In this particular sexual system, plant individuals can choose sexual expression according to environmental conditions. In different years, the sex of the same individual can switch between male and female. For example, *Llaydia oxycarpa* (liliaceae) and *Fritillaria montana* (Peruzzi et al., 2012; Niu et al., 2017). Existing studies showed that some plants with variable sex expression is considered to follow the size-dependent hypothesis, that is, the size of individual plants may determine the sex expression of plants in different growing seasons and years (Zhang et al., 2014; Lindh, 2017; Niu et al., 2017; Astuti et al., 2020). Variability of sex expression is a key survival strategy in the life history of a few plants. Studies have shown that the reproductive success of sessile plants depends heavily on their ability to respond to environmental changes (Goodnoe and Hill, 2018). If plants can choose more effective sexual functions according to environmental changes and can change sex expression with environmental changes, then this sexual strategy is the most adaptive (Zhang et al., 2014; Niu et al., 2017; Blake-Mahmud and Struwe, 2019). At present, the study of plant gender diphasy system has been a hot topic in plant evolutionary biology and plant reproductive ecology.

The response of plants to environmental changes and the potential limitation of essential nutrients makes them allocate resources among important functions (Sardans and Peñuelas, 2015; Goodnoe and Hill, 2018). Any mismatch of resources will affect plant development (Fairhurst et al., 2022). The resources allocation has a trade-off between different functions, and increasing the investment in a certain function requires the expense of other functions (Charnov, 2020). When resources (soil nutrients, water, light and temperature conditions) are limited, excessive reproductive allocation will hinder vegetative growth; overinvestment in reproductive growth without adequate nutritional support may lead to reproductive failure and ultimately affect plant survival (Obeso, 2002). The plasticity of sex expression is considered to be the response of gender resource allocation to environmental conditions (Ashman, 2006; Blake-Mahmud and Struwe, 2020), which is an adaptive strategy for plants to face adverse conditions (Vega-Frutis et al., 2014). At the same time, plants can adapt to environmental changes and improve their survivability by changing phenotypic plasticity of different traits such as plant height, leaf traits and biomass allocation (Zou et al., 2022). Phenotypic plasticity may be a key strategy for small plant populations to continue, and its potential importance is in “buying time” for populations subject to directional environmental change (Nunney, 2016). Therefore, plant phenotypic plasticity expands the adaptability of plants to the environment and enables plants to maintain high fitness over a longer time scale.

In general, sex determination (sex determining genes on sex chromosomes) and sex differentiation (synergistic regulation of environmental factors) jointly determine the sex expression of plants (Golenberg and West, 2013). Although plant sex determination has a strong genetic basis, the individual of the plant can still adjust the sex expression according to the environmental conditions to increase or reduce the investment of different components to maximize the proper sexual reproduction (Du et al., 2021). Studies have shown that the sex expression of gender diphasy plants is regulated by environmental factors. Environmental factors affect plant gene expression, hormone synthesis and other pathways, directly or indirectly involved in the process of plant sex expression (Huang et al., 2022). Generally, higher nutrient levels are beneficial for plants to express female function, while lower soil nutrients are more conducive for plants to express male function (Tibeau, 1936; Bertin, 2007). However, whether the increase of soil nutrients can transform males into females or the decrease of nutrients can transform females into males is not clear.

**Figure 1:**
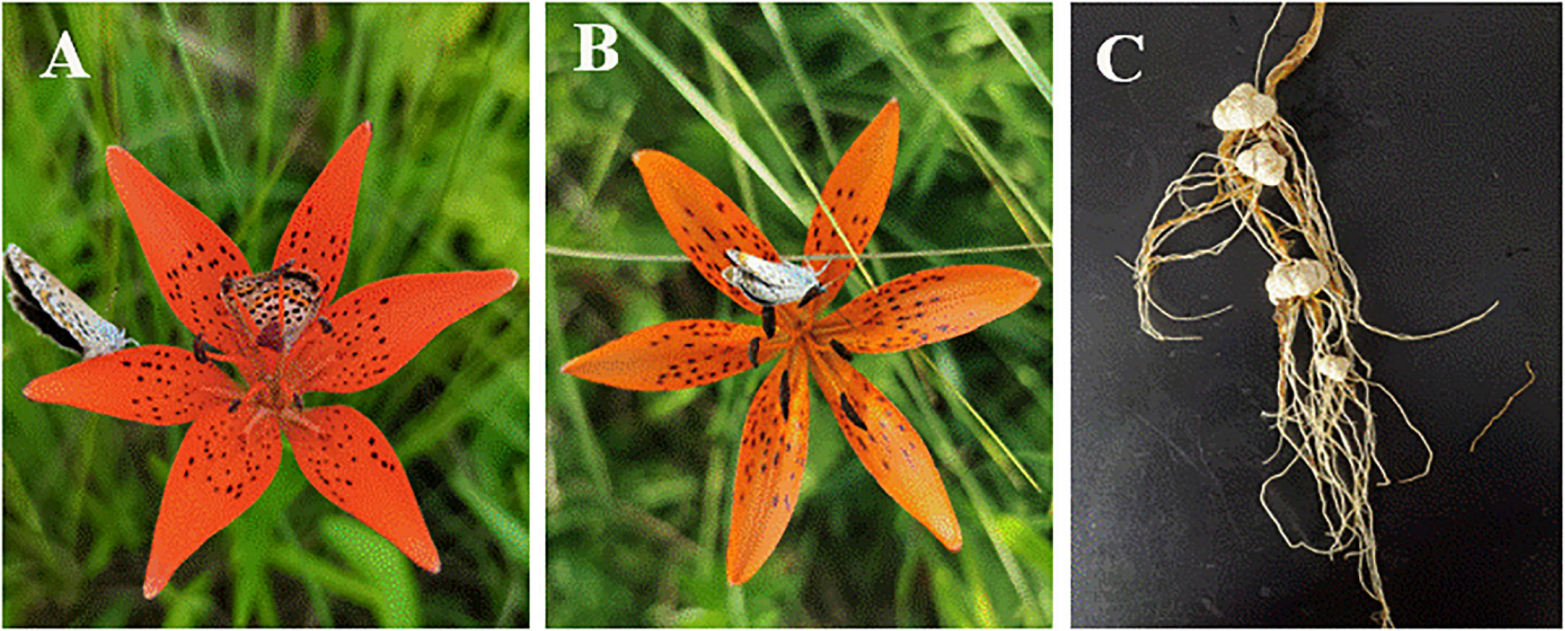
Flowers and bulbs of *Lilium concolor* var. *megalanthum.* **(A)** Hermaphrodite flower, **(B)** male flower, **(C)** bulbs.

*Lilium concolor* var. *megalanthum* is a perennial herb of Liliaceae, mainly growing in wet meadows and peat swamps at an altitude of about 500 m (Du et al., 2021). Its natural population only blooms once a year. Most flowering individuals have one flower, and a few individuals have two or more flowers. According to our previous observations, a large number of plants in the natural population only produce male flowers and coexist with hermaphrodite plants in the same population (**Figure 1**). Before the flowering stage of *Lilium concolor* var. *megalanthum*, male and hermaphrodite plants could not be distinguished morphologically. The individuals having both stamens and pistils are regarded as hermaphrodite plants, that with only stamens or stigma degeneration are regarded as male plants. Studies have found that male flower individuals of *Lilium concolor* var. *megalanthum* act as male parents to transmit genes to the next generation through pollen, while hermaphrodite flower individuals can provide pollen and ovules, act as female parents to transmit genes through ovules, which may be the sexual system of gender diphasy (Du et al., 2021). Whether the *Lilium concolor* var. *megalanthum* is a sexual gender diphasy system needs further verification. In addition, from the current research, it is not clear whether environmental factors can regulate the gender transfer of *Lilium concolor* var. *megalanthum*. Here, we designed a nutrient addition experiment to answer three questions: (1) Whether the *Lilium concolor* var. *megalanthum* has a gender diphasy system; (2) What is the trade-off relationship between growth and reproduction of *Lilium concolor* var. *megalanthum* under limited resources; (3) What is the effect of nutrient level on sex expression.

## 2 Materials and methods

### 2.1 Research sites and materials

The plant sample collection site is located in Jinchuan peatland (42°20′56′′N, 126°22′51′′E) of Longwan National Nature Reserve in Northeast China. The climate of the study site is temperate continental monsoon climate. The average annual temperature is 4.1°C, the average annual precipitation is 704.2 mm, which mostly concentrated in July and August (Zhang et al., 2016). We randomly selected *Lilium concolor* var. *megalanthum* plants in Jinchuan peatland and labeled 200 male plants and 200 hermaphrodite plants as experimental bulb materials in 2020. After the growth of the above-ground part of the plant is completed, the bulbs are excavated and brought back to the laboratory. The bulbs were stored in a refrigerator at 4°C. The bulbs of *Lilium concolor* var. *megalanthum* with good growth and no pests and diseases were selected (from male plants and hermaphrodite plants respectively) and weighed. Bulbs were soaked in carbendazim wettable powder diluent (1: 500) with active ingredient content of 50% for 5 min, disinfected and dried for pot planting. The soil in the pot is turfy soil mixed with farmland soil (volume ratio of 1: 1). The bulb was buried upward in the soil substrate, and the top of the bulb was buried 5 cm below the soil surface and slightly compacted to make the bulb in close contact with the soil. The bulbs overwinter and dormancy in the ground, then sprout and gradually emerge into seedlings in 2021.

### 2.2 Experimental design and implementation

This experiment was conducted in 2021. We measured soil nutrient status in different habitats (**Table S1**). Combined with the soil matrix configuration ratio, it was calculated that the application of nitrogen (1.8 g/per pot), phosphorus (120 mg/per pot), and potassium (300 mg/per pot) fertilizers to the *Lilium concolor* var. *megalanthum* plants derived from the hermaphrodite flower bulbs made the soil bottom value level in the basin equal to and lower (CK: no fertilizer application) than the soil bottom value level in the field habitat. The application of fertilizer (3.6 g/per pot), phosphorus (240 mg/per pot) and potassium (600 mg/per pot) to the lily plants from the bulbs of male flowers made the soil bottom value in the pot higher than and equal to (CK: nitrogen: 1.8 g/per pot; phosphorus: 120 mg/per pot; potassium: 300 mg/per pot) the soil base value in the field habitat. Single (N, P, K) and combined (NP, NK, PN, NPK) fertilization methods were used. Urea was selected as nitrogen fertilizer, sodium dihydrogen phosphate as phosphorus fertilizer and potassium sulfate as potassium fertilizer. There were 16 treatments for male flower bulbs and hermaphrodite flower bulbs, and 10 plants for each treatment (single plant in single pot). Bulbs of about 2 g were selected in different treatment combinations (**Table S2**). The bulbs from hermaphrodite plants were larger than those from male plants, but there was no significant difference in bulbs between different treatments.

Fertilization was carried out at the seedling stage (seedling height 20 cm) of *Lilium concolor* var. *megalanthum*. Nitrogen, phosphorus and potassium fertilizers were applied twice before flowering, with the interval of 10 days. The fertilization method was to evenly apply the dilute solution to the root of the plant. The experiment was carried out in Longwan Ecological Experimental Station of Key Laboratory of Wetland Ecology and Vegetation Restoration. The climatic conditions of the site were the same as those of the plant sample collection site (about 500 m away).

### 2.3 Determination and calculation of indexes

The growth and reproduction indexes were measured at the flowering stage. The number of flowers per plant and the sex of flowering plants were recorded, and the biomass of plants was measured after the growth of the aboveground part finished. All plants bloomed one flower in 2021. Sexual fecundity index = flower dry weight/total biomass, asexual fecundity index = bulb dry weight/total biomass. The plant height of single plant was used as an index to measure the plant size. To avoid damaging sampling of bulbs, bulb biomass (dry weight) was calculated by linear fitting function using field survey sampling data. Thirty plants of two sex phenotypes were randomly selected from Jinchuan peatland in mid-July 2020 (full flowering stage). The bulbs were placed in an oven at 105°C for 0.5 h, then dried at 80°C until constant weight. The fresh weight and dry weight (biomass) of bulbs were weighed using a balance (accuracy is 0.001 g). The obtained data are fitted by variables, and the fitting functions are as follows: The regression relationship between dry weight and fresh weight of hermaphrodite flower bulbs is: y=0.3379x-0.0661, R^2^ = 0.91, P<0.001; the regression relationship between dry weight and fresh weight of male flower bulbs is: y=0.271x+0.0241, R^2^ = 0.87, P<0.001. y is dry weight, x is fresh weight.

### 2.4 Data analysis

The independent sample T test was used to analyze the difference in size of bulbs of *Lilium concolor* var. *megalanthum* from hermaphrodite flowers and male flowers. One-way analysis of variance and Duncan multiple comparison test (data conforming to normal distribution and homogeneity of variance) or rank sum test in non-parametric test and Kruskal-Wallis method (data still did not conform to normal distribution and homogeneity of variance after data conversion) were used to test whether there were significant differences in the growth and reproduction indexes of *Lilium concolor* var. *megalanthum* among the different nutrient treatments. The significance was set at 0.05 (α = 0.05). All statistical analyses were performed using SPSS (25.0). All data were plotted using Origin 9.2 software. The data are expressed as mean ± standard error.

## 3 Results

### 3.1 Effect of nutrient on vegetative growth of *Lilium concolor* var. *megalanthum*

#### 3.1.1 Growth traits of *Lilium concolor* var. *megalanthum* derived from hermaphrodite flower bulbs

**Figure 2:**
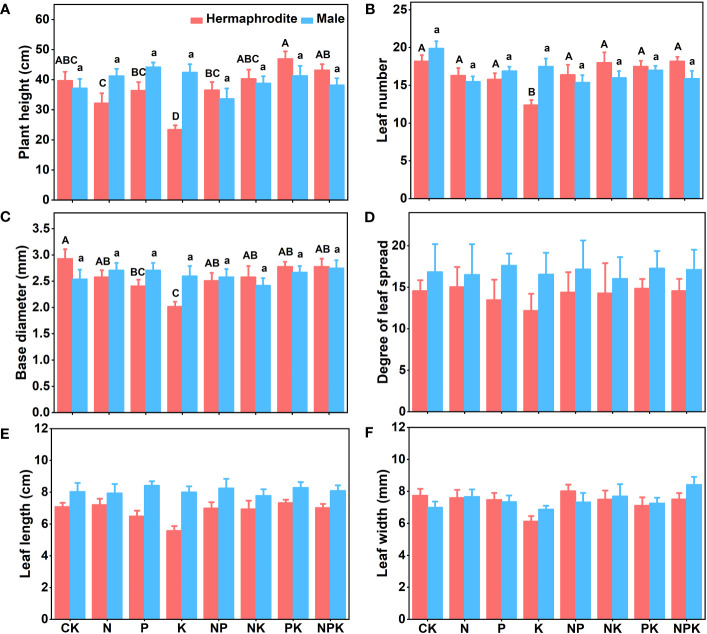
Growth indexes of two sexual phenotypes of *Lilium concolor* var. *megalanthum* under different fertilization treatments in 2021. **(A)**: Plant height; **(B)**: Leaf number; **(C)**: Base diameter; **(D)**: Degree of leaf spread; **(E)**: Leaf length; **(F)**: Leaf width. Different uppercase letters indicated that there was a significant difference between different fertilization treatments (low nutrient addition) of *Lilium concolor* var. *megalanthum* derived from hermaphrodite flower bulbs (P<0.05). Different lowercase letters indicated that there was a significant difference between different fertilization treatments (high nutrient addition) of *Lilium concolor* var. *megalanthum* derived from male flower bulbs (P<0.05).

**Figure 3:**
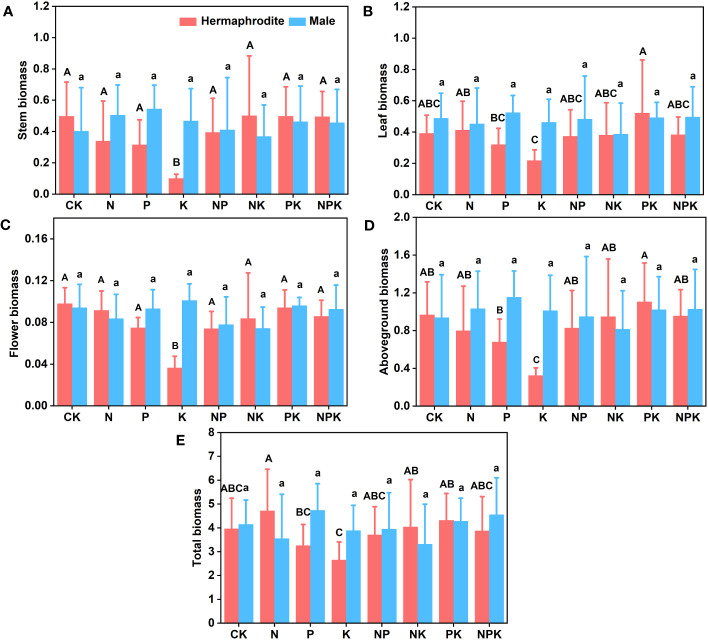
Effects of different fertilization treatments on the biomass of *Lilium concolor* var. *megalanthum* in 2021. **(A)**: Stem biomass; **(B)**: Leaf biomass; **(C)**: Flower biomass; **(D)**: Aboveground biomass; **(E)**: Total biomass.

**Figure 4:**
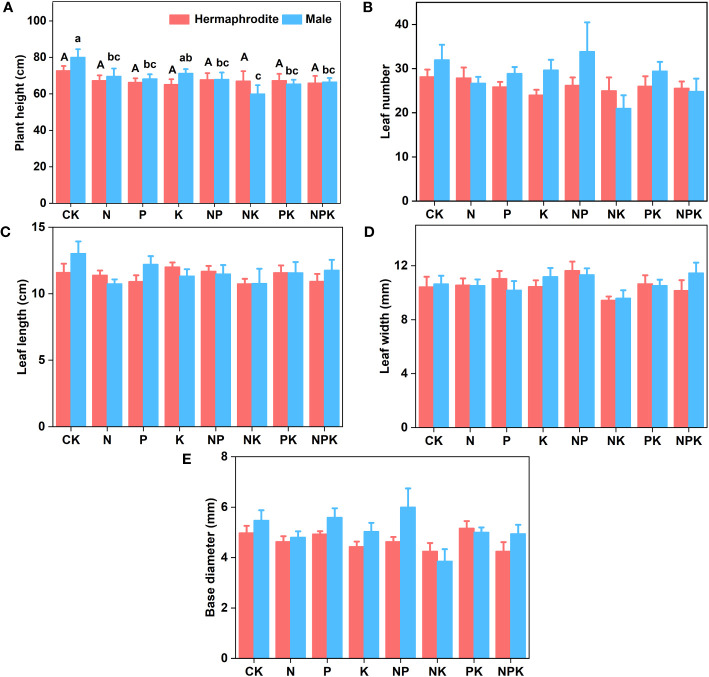
Growth indexes of two sexual phenotypes of *Lilium concolor* var. *megalanthum* under different fertilization treatments in 2022. **(A)**: Plant height; **(B)**: Leaf number; **(C)**: Leaf length; **(D)**: Leaf width; **(E)**: Base diameter.

**Figure 5:**
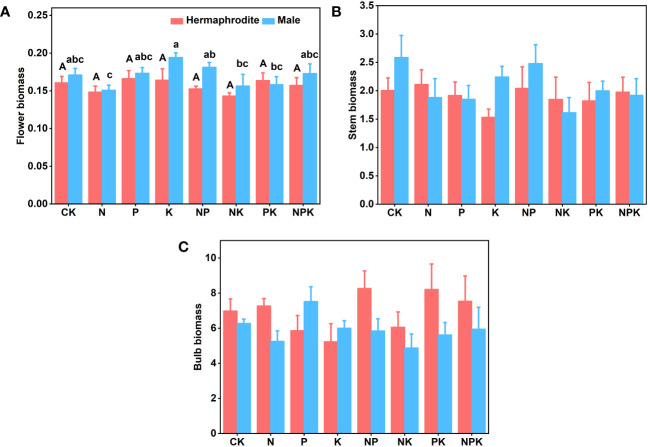
The biomass of two sexual phenotypes of *Lilium concolor* var. *megalanthum* under different fertilization treatments in 2022. **(A)**: Flower biomass **(B)**: Stem biomass; **(C)**: Bulb biomass.

Under different nutrient levels, there were differences in the growth indexes of *Lilium concolor* var. *megalanthum* from the bulbs of hermaphrodite plants in 2021. In the potassium addition treatment, the plant height (P<0.001) and leaf number (P<0.01) of *Lilium concolor* var. *megalanthum* were significantly lower than those of other treatment groups (**Figures 2A, B**). At the same time, its base diameter (P<0.01) was also significantly reduced (**Figure 2C**). There were no significant differences in degree of leaf spread, leaf length and leaf width (P>0.05) among these treatment groups (**Figures 2D–F**). In the potassium addition treatment, the stem biomass (P<0.01), flower biomass (P<0.01) and aboveground biomass (P<0.01) of *Lilium concolor* var. *megalanthum* were significantly lower than those of other treatment groups (**Figures 3A, C, D**). At the same time, its leaf biomass (P<0.05) and total biomass (P<0.05) were also significantly lower (**Figures 3B, E**). There were no significant differences in bulb biomass, fibrous root biomass, rhizome biomass and belowground biomass among these treatment groups (P>0.05, **Figures S1A–D**). There were no significant differences in plant height, leaf number, leaf length, leaf width and biomass among different treatment groups in 2022 (P>0.05, **Figures 4A–E**, **5A–C**).

#### 3.1.2 Growth traits of *Lilium concolor* var. *megalanthum* derived from male flower bulbs

There were no significant differences in plant height, leaf number, base diameter, leaf length, leaf width and biomass (P>0.05) between different treatments of *Lilium concolor* var. *megalanthum* derived from male flower bulbs under higher nutrient addition treatment in 2021 (**Figures 2A–F**, **3A–E**). Except for the potassium addition treatment group, the plant height (P<0.05) of the other treatment groups decreased significantly than CK in 2022 (**Figure 4A**). The flower biomass of *Lilium concolor* var. *megalanthum* in potassium addition treatment (P<0.05) was significantly higher than that in nitrogen addition treatment (**Figure 5A**). There were no significant differences in leaf number, base diameter, leaf length and leaf width, stem biomass and bulb biomass among different treatments (P>0.05, **Figures 4B–E**, **5B, C**).

### 3.2 Effects of nutrients on reproductive traits of *Lilium concolor* var. *megalanthum*

#### 3.2.1 Reproductive traits of *Lilium concolor* var. *megalanthum* from hermaphrodite flower bulbs

**Figure 6:**
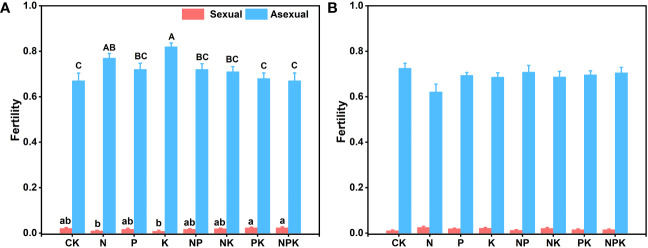
The fecundity index of *Lilium concolor* var. *megalanthum* in 2021. **(A)**: *Lilium concolor* var. *megalanthum* from hermaphrodite flower bulbs **(B)**: *Lilium concolor* var. *megalanthum* from male flower bulbs. Different lowercase letters indicated that there was a significant difference in sexual fecundity between different fertilization treatments (P<0.05). Different capital letters indicated that there was a significant difference in asexual fecundity between different fertilization treatments (P<0.05).

Different nutrient addition treatments had little effects on reproductive traits of *Lilium concolor* var. *megalanthum* from the bulbs of hermaphrodite plants. In the potassium addition treatment group, the flower diameter of *Lilium concolor* var. *megalanthum* was significantly smaller than that of other treatment groups (P<0.01, **Table 1**). In addition, the asexual fecundity (P<0.01) was significantly increased, while the sexual fecundity (P<0.05) was significantly decreased (**Figure 6A**). The flower longevity, petal length, petal width, stamen length, stigma height and ovary height of *Lilium concolor* var. *megalanthum* were not significantly different among nutrient addition treatments (P>0.05) (**Table 1**). In 2022, the number of *Lilium concolor* var. *megalanthum* flowers increased compared with 2021 (all plants have a flower), but there were no significant differences between different treatments (**Table 2**). The stamen length (P<0.05) of *Lilium concolor* var. *megalanthum* was significantly increased under the addition of compound nutrient nitrogen, phosphorus and potassium. There were no significant differences in flower number, flower longevity, petal length, petal width, stigma height and ovary height among different nutrient levels (P>0.05, **Table 2**).

**Table 1:** Reproduction index of *Lilium concolor* var. *megalanthum* derived from hermaphrodite flower bulbs in 2021.

Parameters	CK	N	P	K	NP	NK	PK	NPK
Flower longevity (d)	7.25 ± 0.16a	6.70 ± 0.25ab	6.07 ± 0.38ab	7.25 ± 0.75a	6.13 ± 0.25ab	6.00 ± 0.32b	6.67 ± 0.20ab	6.67 ± 0.42ab
Flower diameter (cm)	6.76 ± 0.38ab	7.28 ± 0.40a	5.31 ± 0.76b	3.55 ± 0.75c	5.80 ± 0.18ab	6.01 ± 0.42ab	7.23 ± 0.43a	6.63 ± 0.27ab
Corolla length (cm)	4.63 ± 0.10a	4.64 ± 0.16a	3.90 ± 0.49ab	3.75 ± 0.05b	4.41 ± 0.15ab	4.42 ± 0.23ab	4.71 ± 0.12a	4.53 ± 0.13ab
Corolla width (mm)	14.12 ± 0.79a	13.39 ± 1.47a	11.77 ± 1.62a	8.44 ± 2.01b	11.59 ± 0.56a	12.64 ± 0.79a	14.20 ± 0.66a	12.90 ± 0.80a
Stamen length (mm)	33.00 ± 1.16a	31.24 ± 0.94a	29.65 ± 1.45ab	26.94 ± 0.33b	31.28 ± 1.14a	30.07 ± 1.20ab	33.52 ± 0.92a	32.26 ± 0.81a
Stigma height (mm)	19.10 ± 0.71a	17.49 ± 0.43a	17.78 ± 1.15a	——	17.02 ± 0.62a	17.97 ± 0.66a	18.75 ± 0.57a	17.12 ± 0.77a
Ovary height (mm)	16.14 ± 0.77ab	16.66 ± 0.96a	14.89 ± 0.58ab	——	14.35 ± 0.59b	16.33 ± 0.74ab	16.53 ± 0.43ab	15.93 ± 0.64ab

Different letters in the same row indicated significant differences between different fertilization treatments (P < 0.05). The same below.

**Table 2:** Reproduction index of *Lilium concolor* var. *megalanthum* derived from hermaphrodite flower bulbs in 2022.

Parameters	CK	N	P	K	NP	NK	PK	NPK
Flower number	2.25 ± 0.25a	2.25 ± 0.31a	1.89 ± 0.26a	1.67 ± 0.17a	2.00 ± 0.30a	2.33 ± 0.47a	1.83 ± 0.17a	1.89 ± 0.26a
Flower longevity (d)	4.37 ± 0.22a	3.92 ± 0.21a	4.03 ± 0.25a	4.31 ± 1.01a	4.21 ± 1.06a	4.03 ± 0.99a	4.2 ± 1.18a	4.08 ± 1.06a
Flower diameter (cm)	8.02 ± 0.27ab	7.77 ± 0.24ab	8.06 ± 0.23ab	8.27 ± 0.28a	8.15 ± 0.26a	8.29 ± 0.23a	8.71 ± 0.37a	7.13 ± 0.29b
Corolla length (cm)	5.90 ± 0.11b	5.74 ± 0.13b	5.85 ± 0.10b	6.34 ± 0.14a	5.93 ± 0.10ab	5.82 ± 0.10a	6.06 ± 0.24ab	5.79 ± 0.22b
Corolla width (mm)	14.67 ± 0.53a	14.65 ± 0.42a	15.34 ± 0.48a	14.87 ± 0.69a	16.13 ± 0.79a	15.07 ± 0.56a	14.94 ± 0.81a	15.66 ± 0.75a
Stamen length mm)	37.77 ± 0.44b	37.51 ± 0.58b	37.51 ± 0.64b	39.18 ± 0.51ab	38.80 ± 0.54ab	38.70 ± 0.62ab	38.56 ± 0.51ab	40.44 ± 1.22a
Stigma height (mm)	21.01 ± 0.59ab	19.76 ± 0.62ab	19.19 ± 0.97b	21.78 ± 0.46a	21.09 ± 0.59ab	21.65 ± 0.31a	20.01 ± 0.64ab	20.84 ± 1.30ab
Ovary height (mm)	21.24 ± 0.54ab	21.58 ± 0.57ab	22.98 ± 0.66a	22.48 ± 0.75ab	20.85 ± 0.53b	20.97 ± 0.39ab	21.84 ± 0.55ab	22.88 ± 1.01ab

#### 3.2.2 Reproductive traits of *Lilium concolor* var. *megalanthum* derived from male flower bulbs

There were no significant differences in flower longevity, flower diameter, petal width, stigma height, ovary height, sexual reproduction and asexual reproduction of *Lilium concolor* var. *megalanthum* from male flower bulbs among different nutrient levels in 2021 (P>0.05, **Table 3**; **Figure 6B**). The petal length (P<0.05) and stamen length (P<0.01) were significantly decreased in nitrogen and potassium addition treatments than other nutrient addition treatments (**Table 3**). The petal length of *Lilium concolor* var. *megalanthum* in the CK increased significantly in 2022 (P<0.001, **Table 4**). The ovary height in nitrogen addition treatment decreased significantly than that of CK (P<0.05, **Table 4**). There were no significant differences in the number of flowers, flower longevity, petal width and stigma height of *Lilium concolor* var. *megalanthum* among different treatments (P>0.05, **Table 4**).

**Table 3:** Reproduction index of *Lilium concolor* var. *megalanthum* derived from male flower bulbs in 2021.

Parameters	CK	N	P	K	NP	NK	PK	NPK
Flower longevity (d)	5.60 ± 0.29a	6.56 ± 0.24a	5.61 ± 0.20a	5.44 ± 0.52a	6.29 ± 0.26a	6.19 ± 0.30a	5.42 ± 0.61a	6.56 ± 0.40a
Flower diameter (cm)	7.12 ± 0.63a	6.62 ± 0.25a	6.94 ± 0.31a	7.03 ± 0.61a	6.93 ± 0.37a	6.56 ± 0.37a	6.79 ± 0.39a	6.65 ± 0.33a
Corolla length (cm)	4.94 ± 0.18a	4.27 ± 0.17bc	4.60 ± 0.12ac	4.75 ± 0.22ac	4.41 ± 0.17ac	4.20 ± 0.17c	4.81 ± 0.17ab	4.73 ± 0.20ac
Corolla width (mm)	12.70 ± 0.45a	12.93 ± 0.70a	11.94 ± 0.70a	12.56 ± 0.72a	12.14 ± 0.59a	11.74 ± 0.78a	12.15 ± 0.61a	12.89 ± 0.83a
Stamen length (mm)	33.45 ± 0.85a	29.18 ± 0.94b	32.90 ± 0.74a	33.25 ± 0.98a	31.02 ± 1.14ab	28.80 ± 0.96b	32.22 ± 1.13a	31.68 ± 0.77ab
Stigma height (mm)	16.35 ± 1.97a	15.74 ± 0.52a	18.12 ± 0.41a	17.10 ± 0.57a	17.09 ± 1.19a	16.72 ± 0.50a	17.65 ± 0.72a	17.70 ± 0.95a
Ovary height (mm)	12.76 ± 2.66b	14.58 ± 0.58ab	15.70 ± 0.77ab	15.40 ± 0.69ab	14.79 ± 1.34ab	14.50 ± 0.68ab	15.66 ± 0.71ab	16.81 ± 0.71a

**Table 4:** Reproduction index of *Lilium concolor* var. *megalanthum* derived from male flower bulbs in 2022.

Parameters	CK	N	P	K	NP	NK	PK	NPK
Flower number	2.38 ± 0.46a	2.67 ± 0.29a	2.00 ± 0.24a	2.10 ± 0.23a	2.00 ± 0.31a	1.67 ± 0.33a	2.29 ± 0.29a	2.00 ± 0.37a
Flower longevity (d)	4.00 ± 0.25ab	3.71 ± 0.18b	3.71 ± 0.33b	3.93 ± 0.27ab	3.77 ± 0.29b	4.147 ± 0.53ab	4.92 ± 0.39a	3.44 ± 0.47b
Flower diameter (cm)	8.65 ± 0.47a	8.02 ± 0.34a	8.07 ± 0.30a	8.61 ± 0.36a	6.78 ± 0.526a	8.21 ± 0.56a	7.71 ± 0.38a	7.55 ± 0.67a
Corolla length (cm)	6.51 ± 0.09a	5.80 ± 0.11cd	6.13 ± 0.12ac	6.35 ± 0.09ab	6.28 ± 0.17ab	5.48 ± 0.24d	5.81 ± 0.15cd	5.60 ± 0.15bc
Corolla width (mm)	15.82 ± 0.39a	14.84 ± 0.47ab	15.68 ± 0.53a	14.57 ± 0.54ab	14.31 ± 0.54ab	14.32 ± 0.84ab	13.64 ± 0.34b	14.72 ± 0.96ab
Stamen length (mm)	37.28 ± 0.40a	37.04 ± 0.40a	38.62 ± 0.66a	37.71 ± 0.40a	36.95 ± 0.17a	36.80 ± 1.03a	36.81 ± 0.58a	36.89 ± 0.94a
Stigma height (mm)	21.63 ± 0.88a	20.43 ± 0.52a	21.40 ± 0.38a	22.08 ± 0.74a	21.72 ± 0.87a	20.66 ± 0.90a	20.91 ± 0.52a	21.42 ± 0.48a
Ovary height (mm)	23.22 ± 1.00a	19.56 ± 0.44b	21.62 ± 0.77ab	22.93 ± 0.76a	22.66 ± 0.93a	21.99 ± 1.17ab	21.37 ± 0.91ab	21.09 ± 0.83ab

### 3.3 Effects of nutrients on sex expression of *Lilium concolor* var. *megalanthum*

Under the conditions that the nutrient level is equal to or lower than that of natural habitats, the plant size of most plants (91.25%) in 2021 is less than that in 2020 (**Table 5**), but the bulbs of most plant (96.25%) are larger than the previous year (**Table S3**). Among them, 60% of the plants continued to produce hermaphrodite flowers, 11.25% of the plants produce male flowers, and 28.75% of the plants did not bloom (**Table 5**). In the treatments of single nutrient element, when the content of phosphorus, potassium, and nitrogen and phosphorus is low, the proportion of plants not flowering or produce male flowers is higher. In the treatments of compound nutrient addition, plants are more inclined to produce hermaphrodite flowers. In 2022, the morphological size and bulb size of all plants were larger than those in 2021, and all plants produced hermaphrodite flowers.

**Table 5:** Effects of different fertilization treatments on sex change of *Lilium concolor* var. *megalanthum* from hermaphrodite flower bulbs.

Treatments	Proportion of plant size variation				Proportion of plants with different flowering types				
	2021		2022		2021			2022	
	Larger	Smaller	Larger	Smaller	Hermaphrodite	Male	Non-flowering	Hermaphrodite	Male
CK	2 (20.00%)	8 (80.00%)	8 (100%)	0(0%)	8 (80.00%)	0 (0.00%)	2 (20.00%)	8 (100%)	0(0%)
N	1 (10.00%)	9 (90.00%)	8 (100%)	0(0%)	5 (50.00%)	0 (0.00%)	5 (50.00%)	8 (100%)	0(0%)
P	0 (0.00%)	10(100.00%)	9 (100%)	0(0%)	5 (50.00%)	2 (20.00%)	3 (30.00%)	9 (100%)	0(0%)
K	0 (0.00%)	10(100.00%)	9 (100%)	0(0%)	0 (0.00%)	2 (20.00%)	8 (80.00%)	9 (100%)	0(0%)
NP	1 (10.00%)	9 (90.00%)	10 (100%)	0(0%)	7 (70.00%)	1 (10.00%)	2 (20.00%)	10 (100%)	0(0%)
NK	1 (10.00%)	9 (90.00%)	9 (100%)	0(0%)	6 (60.00%)	3 (30.00%)	1 (10.00%)	9 (100%)	0(0%)
PK	1 (10.00%)	9 (90.00%)	7 (100%)	0(0%)	8 (80.00%)	1 (10.00%)	1 (10.00%)	7 (100%)	0(0%)
NPK	1 (10.00%)	9 (90.00%)	9 (100%)	0(0%)	9 (90.00%)	0 (0.00%)	1 (10.00%)	9 (100%)	0(0%)
Total	7 (8.75%)	73 (91.25%)	69(100%)	0(0%)	48 (60.00%)	9 (11.25%)	23 (28.75%)	69(100%)	0(0%)

Under the conditions that the nutrient level is equal to or higher than that of the natural habitat, only 36.25% of the plants with male flowers in 2020 have increased in shape and size in 2021 compared with the previous year (**Table 6**), but the bulbs of all plants have increased compared with the previous year (**Table S3**). Among them, 67.5% of the plants produce hermaphrodite flowers, 10% of the plants continued to produce male flowers, and 22.5% of the plants did not bloom (**Table 6**). Under higher nutrient addition treatment, plants are more inclined to produce hermaphrodite flowers. The promotion effect of single nutrient element addition was more significant than that of compound nutrient addition, and no plants produce male flowers. In 2022, the morphological size and bulb size of all plants increased compared with 2021. In the nitrogen and potassium addition treatment group, only one *Lilium concolor* var. *megalanthum* continued to produce male flowers, and all the other plants produce hermaphrodite flowers (**Table 6**).

**Table 6:** Effects of different fertilization treatments on sex change of *Lilium concolor* var. *megalanthum* from male flower bulbs.

Treatments	Proportion of plant size variation				Proportion of plants with different flowering types				
	2021		2022		2021			2022	
	Larger	Smaller	Larger	Smaller	Hermaphrodite	Male	Non-flowering	Hermaphrodite	Male
CK	3 (30.00%)	7 (70.00%)	8 (100%)	0(0%)	5 (50.00%)	1 (10.00%)	4 (40.00%)	8 (100%)	0(0%)
N	3 (30.00%)	7 (70.00%)	9(100%)	0(0%)	8 (80.00%)	0 (0.00%)	2 (20.00%)	9 (100%)	0(0%)
P	4 (40.00%)	6 (60.00%)	9 (100%)	0(0%)	9 (90.00%)	0 (0.00%)	1 (10.00%)	9 (100%)	0(0%)
K	6 (60.00%)	4 (40.00%)	10 (100%)	0(0%)	7 (70.00%)	0 (0.00%)	3 (30.00%)	10 (100%)	0(0%)
NP	5 (50.00%)	5 (50.00%)	7 (100%)	0(0%)	9 (90.00%)	0 (0.00%)	1 (10.00%)	7 (100%)	0(0%)
NK	4 (40.00%)	6 (60.00%)	6 (100%)	0(0%)	5 (50.00%)	3 (30.00%)	2 (20.00%)	5 (83.3%)	1(16.7%)
PK	3 (30.00%)	7 (70.00%)	7 (100%)	0(0%)	5 (50.00%)	2 (20.00%)	3 (30.00%)	7 (100%)	0(0%)
NPK	1 (10.00%)	9 (90.00%)	6 (100%)	0(0%)	6 (60.00%)	2 (20.00%)	2 (20.00%)	6 (100%)	0(0%)
Total	29 (36.25%)	51 (63.75%)	62(100%)	0(0%)	54 (67.50%)	8 (10.00%)	18 (22.50%)	61(98.4%)	1(1.6%)

## 4 Discussion

### 4.1 Response of growth traits of *Lilium concolor* var. *megalanthum* to nutrient conditions

The phenotypic plasticity of plants reflects their adaptability to the environment (Solé-Medina et al., 2022). *Lilium concolor* var. *megalanthum* has a variety of reproductive patterns, so its phenotypic plasticity is more diversified than other plants. The plant height of *Lilium concolor* var. *megalanthum* show strong morphological plasticity. In the soil with low nitrogen and phosphorus content, the plant height of *Lilium concolor* var. *megalanthum* derived from bulbs of plants with hermaphrodite flower is significantly reduced. When nutrient resources are constrained, *Lilium concolor* var. *megalanthum* allocates more resources to the leaves to improve the efficiency of photosynthesis and meet the needs of its own growth. For *Lilium concolor* var. *megalanthum* plants derived from bulbs of male plants, higher levels of nutrients enable it to obtain sufficient nutrients, and the resource allocation of each organ changes accordingly during growth and development to achieve maximum fitness, resulting in no significant difference in each growth index between different treatments.This result indicated that there is a resource allocation trade-off for plants in the process of using limited resources, with increasing the resource allocation of some functions while reducing the resource allocation of other functions (Elle, 1999; Ronsheim and Bever, 2000), and this trade-off is variable with different soil nutrient levels.

The accumulation and allocation of plant biomass reflects the way of plant growth and metabolism and affects the functional attributes of plant organs (Hecht et al., 2019). Plants can better capture and preserve limited resources in soil by changing their aboveground and belowground biomass accumulation (Wang et al., 2019). As the main supporting and photosynthesis organs, the stems and leaves of *Lilium concolor* var. *megalanthum* occupy a large biomass allocation during the whole growth period, which is the guarantee for the transportation of flowering and fruiting resources (Russo et al., 2022). However, there was no significant differences in the accumulation of aboveground and belowground biomass of *Lilium concolor* var. *megalanthum* plants derived from bulbs of hermaphrodite flower and male flower plant among the different fertilization treatment groups (except potassium addition treatment). The results showed that *Lilium concolor* var. *megalanthum* considered the dual needs of belowground root space expansion and aboveground leaves to obtain light energy resources, and promoted the simultaneous growth of aboveground and belowground parts (Bai et al., 2020). In addition, the response of plants to the potential limitation of essential nutrients makes them reallocate resources based on the trade-off of important organ resource demand (Goodnoe and Hill, 2018). When the content of nitrogen and phosphorus in the soil was low (potassium addition treatment), the *Lilium concolor* var. *megalanthum* plants derived from bulbs of hermaphrodite flower plant reduced the input of aboveground biomass and increased the accumulation of belowground biomass. *Lilium concolor* var. *megalanthum* allocated most of the resources to the belowground part, mainly to promote the growth of bulb and accumulation of bulb biomass. The bulbs of the mother plant accumulate more resources for the growth of the next year, which may be a reproductive strategy to remedy for the low efficiency of sexual reproduction of aboveground parts. Secondly, it may also be that sexual reproduction requires a lot of resource input, and the resource input of asexual reproduction is lower than that of sexual reproduction. Therefore, the selection of asexual reproduction may make the population diffusion efficiency and reproductive success rate higher.

### 4.2 Response of reproductive traits of *Lilium concolor* var. *megalanthum* to nutrient treatment

The uptake and accumulation of nutrients in different organs can indirectly reflect the nutritional needs of plants at a certain stage (Quiñones et al., 2012). At the same time, the lack or excess of one or more nutrients may cause physiological stress and hinder the growth and development of plants (Srivastava, 2013). We found that the flower diameter of *Lilium concolor* var. *megalanthum* derived from bulbs of hermaphrodite flower plant decreased significantly under low soil nitrogen and phosphorus (potassium addition treatment) nutrient levels; but the flower diameter increased significantly under the condition of nitrogen addition. This shows that nitrogen can promote the growth of flower of *Lilium concolor* var. *megalanthum* plants, and larger flowers production has the advantage of attracting pollinators and improving their fitness. However, the petal length and stamen length of *Lilium concolor* var. *megalanthum* derived from bulbs of male flower plants were significantly reduced at higher nitrogen levels. It can be thought that the flowers of the plant are sensitive to nutrient levels, and excessive nutrient levels will inhibit them. In addition, the flower diameter of *Lilium concolor* var. *megalanthum* decreased while the flower longevity was longer. This reflects the trade-off between flower longevity and flower size. Limited resource allocation restricts plants from either producing larger flowers or having longer flowering period. Adaptability under low resource conditions may optimize resource allocation by limiting the size of flowers at the expense of extending flower longevity. Our results only showed the trade-off between flower longevity and flower size, but many studies have demonstrated the trade-off between flower longevity and flower number, and between flower size and flower number (Caruso et al., 2012; Spigler and Woodard, 2019). This is a reproductive strategy formed by plants under adverse environmental conditions, which is the embodiment of their adaptation to environmental changes.

*Lilium concolor* var. *megalanthum*, as a perennial herb, can not only produce bulbs to form ramets through clonal growth, but also produce seeds through sexual reproduction for germinating and forming new seedlings. This may lead to trade-off between the organs of the plant itself when competing for limited resources, including the trade-off between asexual and sexual reproduction. We found that the higher asexual fecundity of *Lilium concolor* var. *megalanthum*, the lower sexual fecundity, which indicates that the fecundity of *Lilium concolor* var. *megalanthum* has great ecological plasticity. Long-term asexual reproduction will reduce the genotype of its population. Once the environment changes, *Lilium concolor* var. *megalanthum* population will face great risks. The *Lilium concolor* var. *megalanthum* population can survive well, may be dependent on the supplement of sexual reproduction to asexual reproduction (Arizaga and Ezcurra, 2002). A large number of flowering can increase the opportunity for gene exchange between populations and produce genotypes that are more adaptable to changing environments. This trade-off mechanism of *Lilium concolor* var. *megalanthum* makes its population easy to adapt to the environmental conditions in the region, which is conducive to the maintenance of its population.

Our results showed that when the availability of resources is limited, there is a strong trade-off between the different functions of *Lilium concolor* var. *megalanthum*. However, the effect of soil nutrient difference on resource allocation of different functions was not significant in the second year. In the first year of the growing season, all *Lilium concolor* var. *megalanthum* plants produce one flower, while in the second year, most plants produce two or three flowers, and some individuals even produce 5-6 flowers. The number of flowers proves that *Lilium concolor* var. *megalanthum* have sufficient nutrients for sexual reproduction (Henriot et al., 2019). We believed that the reason may be that the bulbs of *Lilium concolor* var. *megalanthum* provide sufficient resources for sexual reproduction, and the resource allocation of different organs is less regulated by soil nutrients.

### 4.3 Effects of nutrients on sex expression in *Lilium concolor* var. *megalanthum*

We observed the sex expression of *Lilium concolor* var. *megalanthum* plants for three consecutive growing seasons, and found that under the nutrient levels lower than and equal to the natural habitat, the size of the majority of plants in the second growing season decreased, but bulbs increased after *Lilium concolor* var. *megalanthum* consumed more resources to produce hermaphrodite flowers, and some plants changed sex to produce male flowers or did not bloom. Zhang et al. (2014) also found that *Lilium apertum* (Liliaceae) can switch from one gender stage to another with plant size changes from year to year. This result is consistent with our findings. The availability of resources affects the size of plants, indicating that nutrient levels play a key role in determining plant sex expression. Some plants not expressed as hermaphrodite plants for two consecutive growing seasons may be related to certain hormone levels. Many studies have found that many hormones are involved in and regulate plant sex determination. For example, jasmonic acid can promote the differentiation of female flowers (Li et al., 2021), and cytokinin can transform *Vitis vinifera* from male to hermaphrodite flowers (Negi and Olmo, 1966). Studies have shown that high levels of zeatin in hermaphrodite flowers may be an important regulator of sex determination. The difference in zeatin between the two sexual phenotypes is partly due to the difference in the content of available nitrogen as an essential substrate for auxin synthesis (Huang et al., 2022). Auxin can be used as a remote signal to transmit plant rhizosphere nitrogen status and is positively correlated with available nitrogen (Kamada-Nobusada et al., 2013). Therefore, nitrogen deficiency may lead to insufficient auxin synthesis and induce the transformation of hermaphrodite plants to male or vegetative plants (non-flowering) during the second growing season (Huang et al., 2022). This indicates that nutrient levels can affect sex expression by regulating plant hormone synthesis. In our study, when the soil nitrogen and potassium or nitrogen and phosphorus content is low, the proportion of non-flowering *Lilium concolor* var. *megalanthum* plants is more than 50%. We speculate that various nutrient elements produce synergistic effects, jointly regulate hormone synthesis under the condition of limited resources, and regulate plant selective expression the most effective sexual function of reproduction.

The plants with male flowers mainly promote the growth of belowground parts and accumulate belowground resources when the nutrient level was higher than that of natural habitats. In the second growing season, the size of some plants increased, the bulbs of all plants increased, and most plants produce hermaphrodite flowers. Male flowers often have underdeveloped and nonfunctional female organs, which is caused by the stop of pistil development in the late stage of development (Astuti et al., 2020). Contrary to the role of auxin, jasmonic acid was found to play an important role in promoting pistil abortion. The related genes involved in jasmonic acid signal transduction are highly expressed in male plants, eliminating pistil primordia through cell death and promoting male development (Acosta et al., 2009). Our results showed that the increase of single nutrient was more likely to promote the occurrence of hermaphrodite flowers in *Lilium concolor* var. *megalanthum* compared with the increase of compound nutrients. Studies have shown that higher nutrient levels may promote the production of auxin. Auxin may act as an antagonist of jasmonic acid signal transduction related gene expression and play an important regulatory role in plant development and sex expression (male flower female organ abortion) (Nitschke et al., 2016).

In the third growing season, regardless of whether it was originally derived from bulbs of hermaphrodite plants or bulbs of male plants, all plant size and bulbs increased. Most of plants produce male flowers in the first two growing seasons produce hermaphrodite flowers in the third growing season. The results showed that the sex expression of *Lilium concolor* var. *megalanthum* was flexible and regulated by resource availability. When resources in the environment become constrained, the nutrient supply of the *Lilium concolor* var. *megalanthum* does not meet its hermaphrodite flowers, plants will produce male flowers or do not bloom. In this process, bulbs grow and accumulate resources. We found that the sex expression of *Lilium concolor* var. *megalanthum* changed for three consecutive years, and it can be defined as a gender diphasy system. The sex expression is dependent on the size of the bulb (Niu et al., 2017). We speculate that the bulb size is more closely related to the sex expression than the plant size. When the bulb grows to a certain size, and enough to maintain its sexual reproduction, especially female function, plant will always produce hermaphrodite flowers. At this time, the role of resource availability in the environment will decline. This may be a way for resource availability as an environmental factor to affect the sex expression of gender diphasy plants.

## 5 Conclusions

In this study, we confirmed the sex expression variability of *Lilium concolor* var. *megalanthum* plants from year to year, and identified it as a gender diphasy plant. Under the condition of limited resources, there is a trade-off between resource allocation to different functions of plants. This trade-off strategy allows *Lilium concolor* var. *megalanthum* to change their sex expression in adverse environments and choose to express the most effective sexual functions of reproduction. The sex expression strategy of *Lilium concolor* var. *megalanthum* reflects its response to environmental changes and is an ecological adaptation mechanism formed in the long-term evolution process.

## Data availability statement

The raw data supporting the conclusions of this article will be made available by the authors, without undue reservation.

## Author contributions

XC: Conceptualization, data curation, formal analysis, investigation, methodology, validation, visualization, writing—original draft, and writing—review and editing. LW: Investigation and methodology. XY: Funding acquisition, project administration, and resources. ZT: Funding acquisition, project administration, resources, supervision, and writing—review and editing. All authors contributed to the article and approved the submitted version.
