# Supplementary material for: Nutrition regulates sex expression in a gender diphasy plant, Lilium concolor var. megalanthum
Source: Front Plant Sci. 2023 Sep 12;14:1252242. doi: 10.3389/fpls.2023.1252242 (PMC10523294; doi:10.3389/fpls.2023.1252242)
Supplement: Supplementary file 1 [file DataSheet_1.docx]

Supplementary Material

## Supplementary Figures


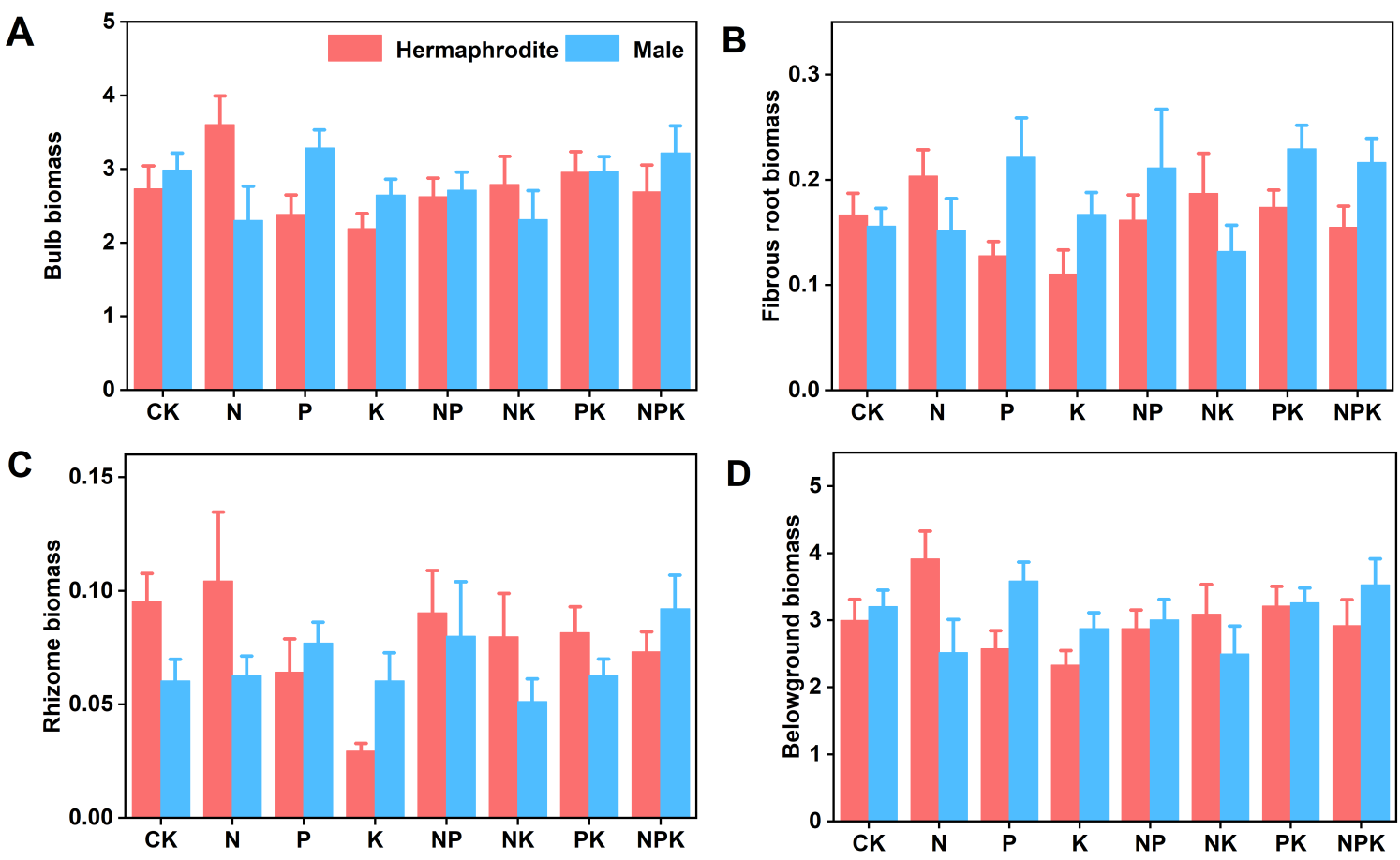


**Supplementary Figure 1.** The effects of different fertilization treatments on two sexual phenotypes of *Lilium concolor* var. *megalanthum* in 2021. (A): Bulb biomass; (B): Fibrous root biomass; (C): Underground stem biomass; (D): Total biomass. There was no significant difference between different treatments.

## Supplementary Tables

**Supplementary Table 1.** Soil nutrient status in different habitats

| Habitats | Available nitrogen mg/kg | Available phosphorus mg/kg | Available potassium mg/kg |
| --- | --- | --- | --- |
| Jinchuan peatland | 840 | 2.17 | 335.87 |
| Pot culture | 385 | 1.21 | 270.13 |

**Supplementary Table 2.** Bulb weights selected for different treatments

| Treatments | Bulb from hermaphrodite plant (g) | Bulb from male plant (g) |
| --- | --- | --- |
| CK | 2.44±0.10aA | 1.93±0.46bA |
| N | 2.16±0.18aA | 1.8±0.46aA |
| P | 2.25±0.20aA | 1.97±0.76aA |
| K | 2.28±0.12aA | 1.80±0.19bA |
| NP | 2.59±0.17aA | 1.80±0.57bA |
| NK | 2.24±0.21aA | 1.84±0.88aA |
| PK | 2.56±0.18aA | 1.83±0.57bA |
| NPK | 2.12±0.17aA | 2.03±0.57aA |

Different lowercase letters in the same row indicate significant differences between two sexual phenotypes, and different uppercase letters in the same column indicate significant differences among different treatments (P<0.05).

**Supplementary** **Table 3.** Effects of different fertilization treatments on the size of bulbs of *Lilium concolor* var. *megalanthum* from hermaphrodite flowers and male flowers

| Treatments | Proportion of hermaphrodite bulb size variation | | | | | | | Proportion of male bulb size variation | | | | |
| --- | --- | --- | --- | --- | --- | --- | --- | --- | --- | --- | --- | --- |
|  | 2021 | | | 2022 | | 2021 | | | | | 2022 | |
|  | Larger | Smaller | Larger | | Smaller | | Larger | | Smaller | Larger | | Smaller |
| CK | 9 (90.00%) | 1 (10.00%) | 8 (100.00%) | | 0 (0.00%) | | 10 (100.00%) | | 0 (0.00%)  0 (0.00%)  0 (0.00%)  0 (0.00%)  0 (0.00%)  0 (0.00%)  0 (0.00%)  0 (0.00%)  0 (0.00%) | 8 (100.00%) | | 0 (0.00%) |
| N | 9 (90.00%) | 1 (10.00%) | 8(100.00%) | | 0 (0.00%) | | 10 (100.00%) | |  | 9 (100.00%) | | 0 (0.00%) |
| P | 10 (100.00%) | 0 (0.00%) | 9 (100.00%) | | 0 (0.00%) | | 10 (100.00%) | |  | 9 (100.00%) | | 0 (0.00%) |
| K | 10 (100.00%) | 0 (0.00%) | 9 (100.00%) | | 0 (0.00%) | | 10 (100.00%) | |  | 10 (100.00%) | | 0 (0.00%) |
| NP | 10 (100.00%) | 0 (0.00%) | 10(100.00%) | | 0 (0.00%) | | 10 (100.00%) | |  | 7 (100.00%) | | 0 (0.00%) |
| NK | 10 (100.00%) | 0 (0.00%) | 9 (100.00%) | | 0 (0.00%) | | 10 (100.00%) | |  | 6 (100.00%) | | 0 (0.00%) |
| PK | 10 (100.00%) | 0 (0.00%) | 7 (100.00%) | | 0 (0.00%) | | 10 (100.00%) | |  | 7 (100.00%) | | 0 (0.00%) |
| NPK | 9 (90.00%) | 1 (10.00%) | 9 (100.00%) | | 0 (0.00%) | | 10 (100.00%) | |  | 6 (100.00%) | | 0 (0.00%) |
| Total | 77 (96.25%) | 3 (3.75%) | 69(100.00%) | | 0 (0.00%) | | 80 (100%) | |  | 62(100.00%) | | 0 (0.00%) |
